# Using science as a differentiator in a crowded digital mental health market

**DOI:** 10.3389/fdgth.2023.1306527

**Published:** 2024-01-08

**Authors:** Jennifer Huberty, Clare C. Beatty, Jacqlyn Yourell

**Affiliations:** Fit Minded, Inc., Phoenix, AZ, United States

**Keywords:** industry, science in business, business strategy, health technology, customers

## Abstract

The digital mental health industry has seen remarkable growth in recent years. However, within this crowded landscape, many companies overlook a critical factor for gaining a competitive edge: the integration of science. In this context, “science” refers to the strategic collection and analysis of information (i.e., data) at digital mental health companies, aimed at guiding business decisions and achieving business objectives. This paper demonstrates that science is integral, yet underutilized in the digital mental health industry, with common misconceptions about its role. When science is integrated within a company, it enables them to (1) innovate, (2) understand customers, (3) make informed decisions, and (4) drive revenue. Digital mental health companies recognizing the multifaceted value of science may be better equipped for sustainable growth and success amid the crowded digital health market.

## 1 Introduction

The digital mental health industry has seen remarkable growth in recent years, with over 20,000 apps now focused on mental wellness (1). This surge has led to a highly competitive market. In order to differentiate themselves, digital mental health companies could consider integrating science into their business strategy (2, 3). The integration of science helps a company demonstrate their innovation and impact on mental health outcomes. Integrating science may include building a science team (external or internal) to collect and analyze data that will inform business decisions, developing a scientific roadmap that supports teams cross-functionally (e.g., product, marketing, science, clinical teams etc.), and/or creating several communication pathways (e.g., presentations, summaries, reports) for translating scientific findings to various key stakeholders (e.g., customers, C-suite, B2B sales). Many companies fail to recognize and harness science's integral role (4, 5) often because they misunderstand how science can be used and integrated in the business context. This lack of understanding can result in missed opportunities. There is a path forward; with education, commitment, and strategy, science can help companies differentiate themselves.

## 2 Misconceptions about science in industry

Drawing from our background as academic researchers who transitioned to industry scientists in the digital mental health sector, we have successfully leveraged science to achieve business goals. This has equipped us with valuable insights into the common misconceptions regarding science's role in business. From these experiences, we have identified four key lessons.

1. **There is no one-size-fits-all scientific approach in business.** For some digital mental health companies, science may manifest as clinical trial studies, while others rely on customer surveys for insights or literature reviews to inform product roadmaps. Science in this context extends beyond peer-reviewed journal articles and clinical trials.
2. **The goals of science in industry are different from academic research.** In industry, science is aligned with business needs, revenue goals, and quarterly key performance indicators (KPIs). Industry science leverages scientific rigor to generate and answer thoughtful questions that advance business goals and secure investments.
3. **Science should not be an afterthought.** Science should be deeply integrated into companies' infrastructure (e.g., internal or external science teams, roadmaps, communication pathways) and operate as a key component of business strategy. Incorporating science into business strategy ensures that decisions are rooted in evidence, which fosters innovation and drives companies towards sustainable growth and success.
4. **Science does not have to be expensive.** The rigor and intensity of scientific endeavors should align with a company's developmental stage. There are instances where companies have disproportionately allocated resources towards scientific pursuits, potentially surpassing the needs of their present phase (e.g., an early-stage startup might not find it beneficial to allocate a significant portion of their budget to science right away) (6). Scientists can educate the C-suite on how to efficiently engage in science, considering the company's stage and short-term business priorities.

## 3 Leveraging science to advance business goals and objectives

As digital mental health companies understand the value of science and how to integrate and conduct science within the context of business, embracing science empowers them to (1) innovate, (2) understand customers, (3) make informed decisions, and (4) drive revenue.

### 3.1 Science for innovation

To maintain a competitive edge, digital mental health companies must identify their core innovation and have a plan to prove it. Science can be used to develop and test innovation and can help answer the question, “What differentiates you from competitors?” This iterative process may include (1) reviewing the scientific literature and competitors' science (if applicable) to understand the landscape and identify opportunities for a competitive edge; (2) formulating and rapidly testing hypotheses for product and service development; (3) iterating based on clinical outcomes to refine offerings; and (4) crafting a compelling narrative that showcases credibility through evidence-based science and unique solutions. In crowded markets, proprietary scientific research is the most reliable way to demonstrate true innovation and attract investors, customers, and talent. As competition increases, digital mental health companies can leverage science to define and defend their competitive edge.

### 3.2 Science to understand customers

Digital mental health companies might assume they know their customers and believe they are effectively addressing their needs/solving their problems. However, without asking scientifically guided questions, companies may fail to truly understand their customers' problems and whether they are effectively addressing needs. This may include identifying specific challenges that customers face or determining why they are using a specific digital mental health product. For example, a digital mental health company that aims to target stress might ask questions, driven by a scientific framework, that determine whether their customers are using their product to help them sleep better (thus potentially eliminating stress). Through this discovery, the company can tailor their solutions to better address the diverse needs of their customers, potentially resulting in enhanced customer satisfaction, increased engagement, and sales.

Many companies use market or user experience (UX) research to understand their customers and facilitate positive user experiences (7). While this can be helpful, market/UX research often focuses on specific problems or features in isolation (8, 9), rather than holistically connecting customer perceptions and experiences to business goals. Additionally, UX research focuses on current users, without considering future implications (9).

There are resource friendly scientific research methods that can be employed to collect more comprehensive insights beyond user experience data alone, providing an evidence-based, outcome-driven methodology. Cross-sectional surveys (which include validated self-report instruments) and focus groups, for example, generate evidence-based insights about customers' needs and preferences, as well as self-reported clinical outcomes that can be used to inform product, content, and pain points in the sales process (10). Additionally, these methods are publishable and can provide early stage companies with preliminary evidence for their products. Ultimately, a scientific approach to understanding the customer may improve engagement, satisfaction, and retention, while simultaneously establishing evidence and credibility.

### 3.3 Science to make informed decisions

Digital mental health companies often make decisions based on their ideas and opinions, without supporting scientific data or evidence (11, 12). This wastes time, money, and resources—all issues avoidable by integrating science from the start, rather than as an afterthought. When embedded early, science can continually inform strategy and decision-making.

Science should be embedded across organizational teams**,** unifying disjointed groups under shared objectives. For example, engineering, product, data science and science teams often work in silos when they should support each other to achieve team objectives. Roadmaps should be built and discussed between teams or developed together. This cross-functional embedding of science enables unified approaches for making company-wide, data-driven decisions that align with evolving business goals and performance indicators. Integrating science cross-functionally helps reveal what is not working for users, such as when outcomes are stagnant. Overall, this helps pinpoint optimal pivot opportunities, guide investments, and illuminate new strategic directions. Integrating science from the onset maximizes its value by steering business strategy and minimizing wasted resources. Business leaders who are committed to long-term success recognize the value science plays in creating an organizational infrastructure.

It is important to note that in the cases where digital mental health companies lack resources to conduct their own science, there are opportunities to form collaborations, create scientific advisory boards, and build partnerships with academic institutions to bridge the gap between science and business needs (13–15). These partnerships may produce the level of evidence and overall credibility that marketing, sales, and investors require for business growth.

### 3.4 Science to drive revenue

Many CEOs express concern that science does not produce *direct* revenue (6); while this may be true, science generates *indirect* revenue (6, 16). Companies often focus on immediate needs, failing to allocate resources that lay their future foundation. Science helps companies build their foundation for the long-term by creating and sustaining the infrastructure that fuels all revenue touch points. There are several key ways science contributes to indirect revenue:

**Investments:** Science builds credibility with both investors and customers by providing proof points that a product works as claimed and is backed by research. Investors need to quickly comprehend why they should fund a company, and science provides the language to convey impact and differentiation. This credibility can help companies secure the investor funding required for continued growth and scaling.
**Sales and Marketing:** Science provides competitive credibility that sales and marketing can leverage to secure partnerships and customers. For instance, promoting research results like, “90% of customers have reduced anxiety after one month,” will educate customers and may help engage and retain them (17). Quantitative results and data appeal to customers and foster trust, as they demonstrate the potential real-world impact that a product or service may have on their lives. Science also equips teams with competitive intel, credibility, and talking points to secure large scale partnerships and business opportunities (because they can cite their own published research studies).
**Customer Connection:** Science helps companies understand their customers to build tailored, personalized offerings (i.e., informed product and content), which improves satisfaction and retention (18–20). For instance, qualitative studies on users of mental health apps can reveal the content or features that users wish to see, providing tangible improvement points to enhance the apps (21, 22). Science also helps attract and acquire customers by showing them tangible results they can expect from using the product or service. Science builds trust as customers realize a company's product delivers on its claims. For wavering buyers, science may be a significant deciding factor (23).
**Business Strategy:** Science supports business strategy by informing decision-making at both the micro (e.g., product development/design) and macro level (e.g., branding/marketing). Science, particularly publications, helps articulate the company's narrative—who they are, how they help customers, and what their strategic vision is for the future.

## 4 Discussion

Science is an integral, yet underutilized asset in the digital mental health industry. Systematic reviews examining digital mental health apps reveal that a mere 2–3.4% substantiate their product and app claims with scientific evidence (24–26). This issue is of paramount significance, not only due to the ongoing mental health crisis and the need for efficacious interventions (27, 28), but also for companies striving to differentiate themselves in a crowded market.

Integrating science has a multifaceted value—from innovation and organizational connectivity, to revenue generation and data points that drive sales. Science provides a way to stand out in a crowded market through innovation and credibility. While science may not directly generate revenue, it is the foundation for all parts of the company that *do* drive revenue. Science and business are fundamentally synergistic. Digital mental health companies that strategically integrate science from the outset and across teams are positioned for sustaining and growing their business above competitors. With strategic vision, executive leadership, and organizational alignment, science can move from an afterthought to a competitive advantage.

## Data Availability

The original contributions presented in the study are included in the article/Supplementary Material, further inquiries can be directed to the corresponding author.
